# HuTAge: a comprehensive human tissue- and cell-specific ageing signature atlas

**DOI:** 10.1093/bioadv/vbaf072

**Published:** 2025-04-03

**Authors:** Koichi Himori, Zhang Bingyuan, Kazuki Hatta, Yusuke Matsui

**Affiliations:** Institute for Glyco-core Research (iGCORE), Tokai National Higher Education and Research System, Nagoya 464-0814, Japan; Institute for Glyco-core Research (iGCORE), Tokai National Higher Education and Research System, Nagoya 464-0814, Japan; Institute for Glyco-core Research (iGCORE), Tokai National Higher Education and Research System, Nagoya 464-0814, Japan; Institute for Glyco-core Research (iGCORE), Tokai National Higher Education and Research System, Nagoya 464-0814, Japan; Biomedical and Health Informatics Unit, Department of Integrated Health Science, Nagoya University Graduate School of Medicine, Nagoya 461-8673, Japan

## Abstract

**Summary:**

Ageing is a complex process that involves interorgan and intercellular interactions. To obtain a clear understanding of ageing, cross-tissue single-cell data resources are required. However, a complete resource for humans is not available. To bridge this gap, we developed HuTAge, a comprehensive resource that integrates cross-tissue age-related information from The Genotype-Tissue Expression project with cross-tissue single-cell information from Tabula Sapiens to provide human tissue- and cell-specific ageing molecular information.

**Availability and implementation:**

HuTAge is implemented within an R Shiny application and can be freely accessed at https://igcore.cloud/GerOmics/HuTAge/home. The source code is available at https://github.com/matsui-lab/HuTAge.

## 1 Introduction

Ageing is widely recognized as a major risk factor for a broad range of diseases, including cardiovascular disease, arthritis, neurodegenerative diseases, and cancer (Franceschi *et al.* 2018). To mitigate these adverse effects and enhance health span, a deep understanding of the molecular mechanisms underlying ageing and its involvement in disease is required. The ageing process involves complex molecular and cellular changes, including dysregulated intercellular communication, epigenetic modifications, transcriptional alterations, genomic instability, and defects in telomere maintenance mechanisms, as evidenced by extensive research (López-Otín *et al.* 2023). These processes are characterized by complex interactions among tissues at the macro level and among cells at the micro level. Given these characteristics, understanding the ageing process requires comprehensive data resources with single-cell resolution that span various tissues.

The Tabula Muris Senis (Tabula Muris Consortium 2020) is a single-cell transcriptomic atlas that spans the entire lifespan of mice and includes data from 23 tissues and organs. This resource is comprehensive and invaluable for examining ageing-related cellular and molecular changes across cell types and tissues. However, comprehensive cross-tissue resources for human ageing research are limited, and a consistent and large-scale single-cell transcriptomic atlas such as the Tabula Muris Senis is lacking. Although studies on ageing phenotypes based on tissue- and cell-specific gene expression in humans exist, these studies often focus on specific cell types within particular tissues (Perez *et al.* 2022, Jeffries *et al.* 2023), which makes systematic comparisons difficult because of differences in the experimental conditions and assay methods used.

The Genotype-Tissue Expression (GTEx) project (GTEx Consortium *et al.* 2020) provides one of the most comprehensive single datasets with the largest variety of tissue types, encompassing RNA-sequencing (RNA-seq) transcriptome profiles of >40 tissues from hundreds of human donors of various ages. Importantly, GTEx has the advantage of including data from multiple tissues from the same individuals. However, while single-cell data are partially included (eight tissues, up to four samples per tissue), they lack sufficient sample size and adequate age-range coverage for ageing research. In contrast, the Tabula Sapiens (Tabula Sapiens Consortium *et al.* 2022) provides cross-tissue single-cell data (24 tissues, up to 13 samples per tissue). Nevertheless, it also has limitations due to the small number of donors and sparse age distribution, making it unsuitable for ageing research when used independently. To address the above limitations, we combined the most comprehensive resources available: GTEx and Tabula Sapiens. One study used the Tabula Muris to deconvolute GTEx data and integrate them both in the context of expression of quantitative trait loci (Donovan *et al.* 2020); however, there have been no studies integrating GTEx and the Tabula Sapiens in the context of ageing. VoyAger (Schneider *et al.* 2024) and GTExVisualizer (Guzz *et al.* 2023) are tissue ageing information resources based on GTEx, but their integration of cellular information is limited (Supplementary Table S1).

Here, we introduce HuTAge, a resource that integrates cross-tissue age-related information from GTEx with the cross-tissue single-cell information from Tabula Sapiens. HuTAge aims to (i) provide comprehensive cross-tissue and single-cell transcriptomic data, (ii) facilitate the identification of age-related molecular signatures across various tissues and cell types, and (iii) enable the exploration of age-dependent changes in gene expression, cell–cell interactions, and transcription factor (TF) activities.

## 2 Implementation

HuTAge is a web-based interface developed using the RStudio R Shiny package (https://github.com/rstudio/shiny), designed for exploring and visualizing tissue- and cell-specific ageing signatures. It can be accessed directly from https://igcore.cloud/GerOmics/HuTAge/home and works on any browser. The user guide for the HuTAge is accessible within the application and through Supplementary Materials of the article. The source code and data are available at https://github.com/matsui-lab/HuTAge.

## 3 Features

### 3.1 Overview

**Figure 1.**
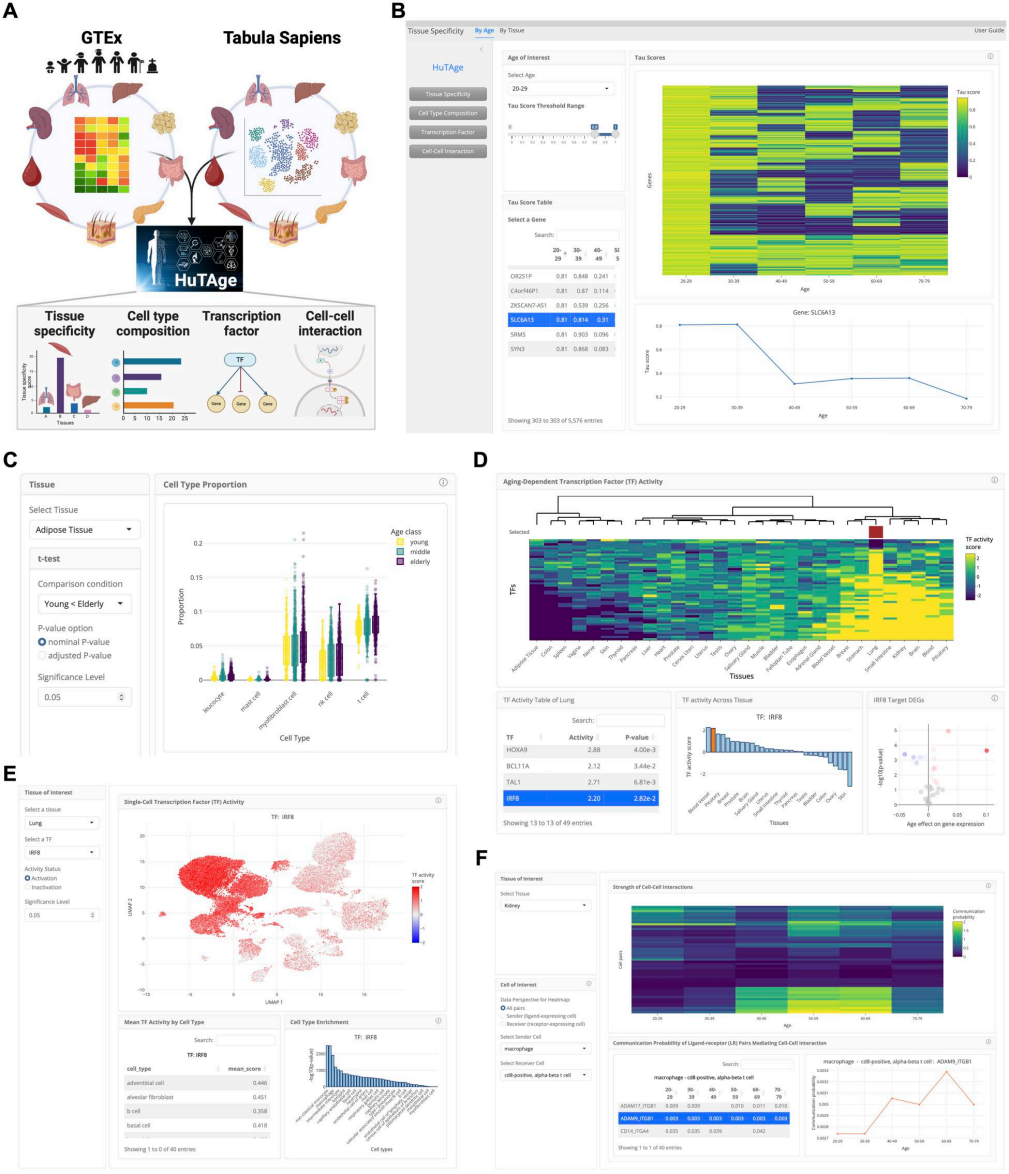
Presentation of HuTAge. (A) Schematic overview of the workflow for exploring tissue- and cell-specific ageing signatures. The web tool HuTAge integrates GTEx bulk organ RNA-seq and Tabula Sapiens single-cell RNA-seq data, providing a comprehensive downstream analysis platform through four distinct modules, as shown below (Created with BioRender.com). (B) Heatmap showing tissue-specificity scores of genes across ages in skeletal muscle. Additionally, users can extract genes with high tissue specificity for selected age groups and browse gene information and age-dependent changes in tables and line plots. (C) Box plot displaying age-related changes in cell type composition estimated from GTEx bulk RNA-seq data. (D) Heatmap showing ageing-related changes in TF activity across tissues. (E) Users can map the activity of selected TFs at the single-cell level using feature plots to visually identify which cell types the TFs are enriched in and quantitatively visualize the enrichment in bar plots. (F) Heatmap displaying age-related changes in cell–cell communication strength, with columns representing age groups and rows representing cell and cell pair combinations. Users can also pro-file the activation scores of ligands and receptors mediating these interactions.

HuTAge comprises bulk RNA-seq expression data from 17 382 samples across 30 normal tissues from individuals spanning the age range of 20–70 years obtained from the GTEx project (v.8) and single-cell RNA-seq expression data from 13 normal tissues provided by the Tabula Sapiens project. To allow interactive exploration of the age-dependent single-cell information across human tissues, we constructed four modules: ‘Tissue specificity’, ‘Cell type composition’, ‘Transcription factor’, and ‘Cell–cell interaction’ (Fig. 1A). The modules are described in detail in the following subsections.

### 3.2 Tissue specificity module

The ‘Tissue specificity’ module enables the investigation of age-dependent changes in the tissue specificity of genes (Fig. 1B), which is assessed by calculating the tissue specificity index (tau) for each gene (Yanai *et al.* 2005, Kryuchkova-Mostacci and Robinson-Rechavi 2017). The ‘By Age’ tab in HuTAge generates heatmaps that display age-dependent changes in the tau scores of genes, calculated for each age group (Fig. 1B). Users can filter the data by selecting a certain age group and adjusting the tau score threshold, allowing for the extraction of tissue-specific genes for the selected age group. The filtered results can be accessed from a table, and when a gene is selected from the table, a line plot showing the age-dependent changes in its specificity score is provided (Fig. 1B). In the ‘By Tissue’ tab, heatmaps show the tau expression fraction scores (assigned to each tissue and indicating the specificity of the given gene for that tissue) of genes for the selected age group, allowing users to identify in which tissues the genes are specifically expressed.

### 3.3 Cell type composition module

The ‘Cell type composition’ module enables the analysis of age-dependent changes in cell type proportions within tissues of interest (Fig. 1C). Cell type proportions were estimated using Bisque (Jew *et al.* 2020) to deconvolute bulk RNA-seq expression data from different age groups in GTEx, using single-cell RNA-seq expression data from Tabula Sapiens as the reference panel. We selected Bisque based on a recent benchmarking study (Huuki-Myers *et al.* 2025) that demonstrated its accurate and robust performance across various RNA extraction protocols. In addition, we used Tabula Sapiens because this comprehensive human single-cell atlas covers multiple tissues, making it particularly suitable for matching the multi-tissue nature of GTEx data. The ‘Cell type composition’ tab outputs box plots and tables showing cell types with significant differences in proportions between any two of three age groups (young, middle-aged, and elderly) within a single tissue (Fig. 1C). Users can select two age groups for comparison and set the significance level. The ‘Cell marker gene’ tab visualizes age-dependent changes in the expression of cell marker genes in selected tissues and cell types in GTEx data using line plots.

### 3.4 Transcription factor module

The ‘Transcription factor’ module is designed to analyse the age dependency of TF activity across tissues (Fig. 1D and E). TF activity was estimated using the decoupleR method (Badia-I-Mompel *et al.* 2022). The ‘Tissue’ tab visualizes age-dependent changes in TF activity across tissues using heatmaps (Fig. 1D). To achieve this, we conducted an age-dependency analysis using ordinal logistic regression based on the R package ordinal (https://github.com/runehaubo/ordinal). The resulting coefficients were used as inputs to estimate TF activity. Users can extract and visualize TFs that have significant age dependency in tissues of interest, and the extracted TFs can be examined in a table at the bottom of the interface. Upon selecting a TF from this table, a bar plot showing its TF activity distribution across tissues is displayed. Furthermore, target gene expression for the selected TF is presented as a volcano plot, reflecting the coefficients and *P*-values from the ordinal logistic regression analysis of the target genes. In the ‘Cell’ tab, based on the tissue and TF selected in the ‘Tissue’ tab, the cellular distribution of TF activity is highlighted in a uniform manifold approximation and projection (UMAP) feature plot (Fig. 1E). Specifically, TF activity values estimated for each single cell in the corresponding tissues from Tabula Sapiens are displayed in the UMAP plot. Users can switch between the activation and inactivation states of TF activity in single cells, as well as adjust the significance level to modify the visualization. The table at the bottom of the interface shows averaged TF activity values for each cell type (Fig. 1E). The bar plot integrates *P*-values from Fisher’s exact test for multiple comparisons, allowing users to identify which cell types are enriched in TF activity (Fig. 1E).

### 3.5 Cell–cell interaction module

The ‘Cell–cell interaction’ module supports the examination of age-dependent changes in cell–cell interaction strength within selected tissues (Fig. 1F). Initially, Bulk-SignalR (Villemin *et al.* 2023) was applied to GTEx bulk RNA-seq data to determine ligand-receptor (L-R) pairs that change with age. In brief, we estimated L-R pairs with significant correlations for each age group within the GTEx bulk RNA-seq data.

These L-R pairs were input into CellChat (Jin *et al.* 2021) along with single-cell RNA-seq data from Tabula Sapiens to estimate age-specific cell–cell interactions. To extract cells that show age-dependent alterations in cell–cell interaction strength, we calculated communication probability between cell types mediated by the input LR pairs for each age group. The ‘Cell–cell’ tab visualizes the strength of interaction between any cell pair within a selected tissue across different age groups in heatmaps (Fig. 1F). By choosing sender or receiver cells of interest, users can access detailed information about the ligands and receptors mediating these interactions. Upon selecting an L-R pair from the table, a line plot showing the interaction probabilities calculated for each age group is displayed (Fig. 1F).

## 4 Comparison with existing tools

Existing tools for tissue ageing analysis and visualization using GTEx data include voyAGEr (Schneider *et al.* 2024) and GTExVisualizer (Guzz *et al.* 2023) (Supplementary table S1). voyAGEr visualizes gene expression changes across 49 human tissues by age and biological sex. In addition to examining age-related alterations in tissue-specific transcriptomes, it analyses co-expressed gene modules that vary with ageing in four specific tissues, enabling enrichment analysis for cell types, biological pathways, and disease associations. In contrast, GTExVisualizer allows users to filter data by gene name and tissue, extract and visualize expression data by age and sex, and test for statistically significant age- or sex-related differences in gene expression. HuTAge distinguishes itself from these tools by integrating both bulk RNA-seq data from GTEx and single-cell RNA-seq data from Tabula Sapiens. While voyAGEr does incorporate some cell-related information, it is limited to four tissues and relies on enrichment analyses of gene co-expression modules and cell-type signatures. Furthermore, HuTAge provides four modules—tissue specificity, cell type composition, transcription factor activity, and cell–cell interaction—thereby enabling a multifaceted exploration of the molecular mechanisms and cellular dynamics of ageing from both bulk and single-cell perspectives. Taken together, these features afford HuTAge a distinct advantage in exploring the biological basis of ageing from multiple angles and at greater depth.

## 5 Case study

**Figure 2.**
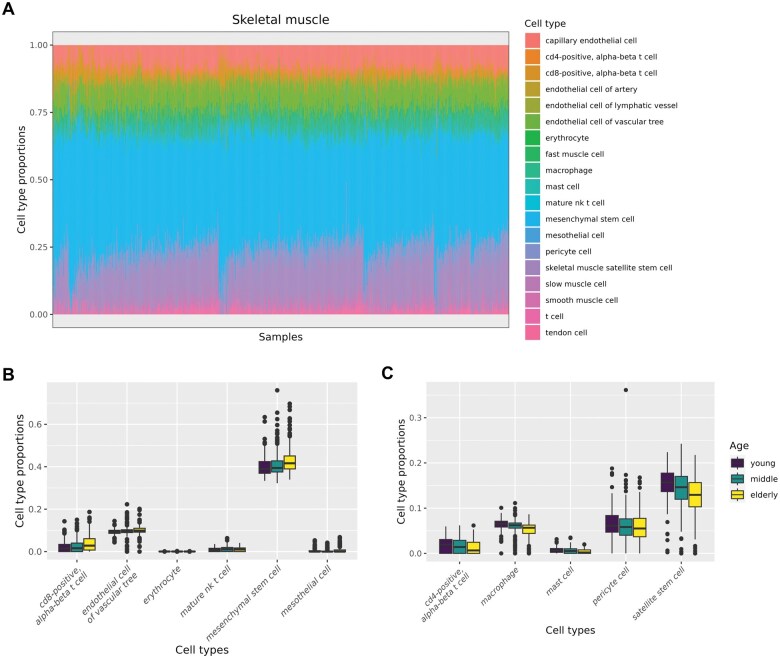
Ageing-associated changes in cell type composition in skeletal muscle. (A) Stacked bar chart of each indicated cell types across skeletal muscle samples (*X*-axis: samples, *Y*-axis: cell type proportion). (B, C) Boxplots showing cell types with significantly higher (B) or lower (C) proportions in elderly individuals compared to young individuals. Three age groups are defined as follows: young (20–39 years), middle (40–59 years), and elderly (60–79 years).

To evaluate the quality of our deconvolution results, we conducted a case study using skeletal muscle tissue bulk RNA-seq data from GTEx and skeletal muscle tissue single-cell RNA-seq data from Tabula Sapiens. From the GTEx dataset, we analysed 803 samples obtained from individuals in their 20–70s. From Tabula Sapiens, we used four samples from individuals in their 30–50s, comprising 30 746 cells classified into 19 cell types. Figure 2A illustrates the proportion of each cell type estimated using the Bisque algorithm across all samples. In skeletal muscle tissue, the proportions of macrophages, muscle satellite cells, CD4+ T cells, mast cells, and pericytes decreased with age, whereas those of CD8+ T cells, mesenchymal stem cells, and vascular endothelial cells increased (Fig. 2B). Few studies have performed single-cell or single-nucleus analyses of skeletal muscle in older adults to investigate how cell type proportions change with ageing (Perez *et al.* 2022, Kedlian *et al.* 2024, Lai *et al.* 2024). The findings from these studies regarding age-related changes in cell type proportions vary, likely because of differences in participant profiles, target muscles, and the marker genes used for annotation. Despite the disparities among studies, the reduction in muscle satellite cells with ageing has been consistently observed. Muscle satellite cells, which are crucial stem cells for skeletal muscle maintenance and regeneration of skeletal muscle, are known to decrease in number with age, and this age-related drop is associated with the deterioration of tissue repair capacity (Brack *et al.* 2005, Chakkalakal *et al.* 2012, Wang *et al.* 2019). In our deconvolution results, we also observed a decrease in the proportion of muscle satellite cells with age, reinforcing the reliability and biological validity of this approach.

## 6 Limitation and future direction

One of the major limitations of our framework is that the ‘Cell’ tab in the ‘Transcription factor’ module does not itself incorporate ageing information. In the ‘Cell type composition’ and ‘Cell–cell interaction’ modules, we designed the framework to evaluate changes in cell composition and intercellular signalling by using both GTEx and Tabula Sapiens data as inputs. In contrast, the ‘Transcription factor’ module conducts GTEx analyses using bulk RNA-seq data and Tabula Sapiens analyses using single-cell RNA-seq data independently. Therefore, the current ‘Cell’ tab is not intended to rigorously assess age-dependent TF activity at the single-cell level but rather to complement tissue-level insights by illustrating the distribution of TFs within individual cells. In the future, when a comprehensive single-cell dataset with sufficient ageing annotations becomes available, this module can be updated to enable more detailed and ageing-focused analysis of TF activity at single-cell level.

## 7 Conclusion

We introduced HuTAge, a comprehensive resource that integrates cross-tissue age-related information from GTEx with cross-tissue single-cell information from Tabula Sapiens. By leveraging the strengths of these comprehensive datasets, HuTAge allows multifaceted and comprehensive cross-tissue and cell-type ageing profiling. It identifies targets with varying degrees of association with and specificity to ageing and interprets this information at the cellular level. This integrated resource offers a comprehensive in silico tool for analysing tissue- and cell-specific ageing gene information in humans. Our web-based interface, developed using the RStudio R Shiny package, allows for interactive exploration and visualization of tissue- and cell-specific ageing signatures. Users can access the platform directly from any browser and perform in-depth analyses using the ‘Tissue specificity’, ‘Cell type composition’, ‘Transcription factor’, and ‘Cell–cell interaction’ modules. We plan to further enhance HuTAge by incorporating additional datasets and expanding its analytical capabilities, focusing on improving user experience, integrating more advanced visualization tools, and ensuring that the platform remains a cutting-edge resource for ageing research. HuTAge demonstrates the effective integration of diverse datasets to address the complexities of ageing, providing researchers with a valuable tool to enhance our understanding of age-related biological processes. This comprehensive and accessible platform aids in the study of gene expression changes across various tissues and cell types, contributing to the advancement of ageing research.

## Supplementary Material

vbaf072_Supplementary_Data

## Data Availability

HuTAge is implemented as a Shiny application and can be accessed at https://igcore.cloud/GerOmics/HuTAge/home. The source code is available at https://github.com/matsui-lab/HuTAge.
