# Supplementary material for: HuTAge: a comprehensive human tissue- and cell-specific ageing signature atlas
Source: Bioinform Adv. 2025 Apr 3;5(1):vbaf072. doi: 10.1093/bioadv/vbaf072 (PMC12005899; doi:10.1093/bioadv/vbaf072)
Supplement: vbaf072_Supplementary_Data [file vbaf072_supplementary_data.zip › SupplementaryMethods.docx]

Supplementary Materials

**HuTAge: a Comprehensive Human Tissue- and Cell-specific Ageing Signature Atlas**

Koichi Himori^1*^, Zhang Bingyuan^1^, Kazuki Hatta^1^, Yusuke Matsui^1,2^

^1^Institute for Glyco-core Research (iGCORE) Tokai National Higher Education and Research System, Nagoya, Japan; ^2^Biomedical and Health Informatics Unit, Department of Integrated Health Science, Nagoya University Graduate School of Medicine, Nagoya, Japan

**Supplementary Table S1.** **Comparison of ageing resources across multiple tissues.**

| **Feature** | **HuTAge** | **VoyAGEr** | **GTExVisualizer** |
| --- | --- | --- | --- |
| **Publication** | This study | Schneider *et al.*, 2024 | Guzz *et al.*, 2023 |
| **Data source (bulk)** | GTEx（30 tissues: broad classification） | GTEx（49 tissues: detailed classification） | GTEx（54 tissues: detailed classification） |
| **Data source (single-cell)** | Tabula Sapiens（13 tissues） | — | — |
| **Integration with single-cell data** | Yes (directly references single-cell data from Tabula Sapiens; 13 tissues) | Limited (limited to ‘cell type enrichment’ using cell type signatures; 4 tissues) | No |
| **Tissue specificity analysis** | Yes (tau-based quantification and cross-tissue heatmaps) | Partial/Yes (cross-tissue heatmaps) | No |
| **Deconvolution analysis** | Yes | No | No |
| **Transcription factor activity analysis** | Yes | No | No |
| **Cell-cell interaction analysis** | Yes | No | No |
| **Co-expression module analysis** | No | Yes (4 tissues) | No |
| **Sex differences analysis and visualization** | No | Yes | Yes |

**‘**—**’** indicates not available

**Supplementary methods**

**Data**

RNA-seq expression data (version 8) from normal tissues were downloaded from The Genotype-Tissue Expression (GTEx) portal (https://gtexportal.org) on October 30, 2023. For the ordinal logistic regression analysis (detailed below), samples were divided into six age groups: 20–29, 30–39, 40–49, 50–59, 60–69, and 70 years. Samples were divided by age into three groups for the cell type proportion analysis (young: 20–29 and 30–39 years; middle: 40–49 and 50–59 years; elderly: 60–69 and 70 years) and into two groups for the analysis of age-related changes in ligand-receptor interactions (young: 20–29 and 30–39 years; elderly: 60–69 and 70 years). Single-cell RNA-seq expression data from normal tissues were downloaded from Tabula Sapiens (https://tabula-sapiens.sf.czbiohub.org/) on December 27, 2023. We further processed the data using Seurat package in R (https://github.com/satijalab/seurat). For the single-cell RNA-seq analysis, only the 13 tissues present in both the GTEx and Tabula Sapiens datasets were included.

**Identification of tissue-specific genes**

Tissue specificity of gene expression was assessed by calculating the tissue specificity index (tau) (Yanai *et al.*, 2004; Kryuchkova-Mostacci and Robinson-Rechavi, 2017). Tau scores were calculated using the calcTau function in the tispec package (https://github.com/BioinfGuru/tispec). To obtain tau scores for each gene within each age group, we first calculated the average expression level of each gene separately for each tissue and age group. Expression values were normalized both within and across tissues prior to tau calculation. A tau score of 1 indicates that a gene is exclusively expressed in one tissue, whereas a score of 0 suggests uniform expression across all tissues. In addition, the calcTau function provides the tissue-specific expression fraction (tef) for each tissue, which quantifies the contribution of each tissue to the overall expression profile of a gene. While tau quantifies the degree of specificity, tef identifies the particular tissue in which the gene is predominantly expressed.

**Inference of cell type composition**

To evaluate ageing-related changes in cell type composition, we deconvoluted GTEx bulk RNA-seq data for each age group using Bisque (Jew *et al.*, 2020). Bisque is a robust method for estimating cell type composition that applies non-negative least squares (NNLS) regression with additional sum-to-one constraints to transformed bulk gene expression data, using paired or unpaired single-cell datasets as references. We selected the Bisque reference-based decomposition approach, using single-cell RNA-seq expression data from Tabula Sapiens as the reference panel. From this scRNA-seq dataset, we extracted the expression matrix and cell type annotations to construct the reference signature matrix. To account for technical biases between sequencing platforms, the Bisque algorithm applies a gene-specific transformation to the bulk RNA-seq data, resulting in a corrected expression matrix that is more comparable to the single-cell reference. Subsequently, the ReferenceBasedDecomposition function was used to deconvolve the bulk RNA-seq data. This approach employs NNLS regression to integrate the transformed bulk expression matrix with the reference signature matrix derived from single-cell RNA-seq data, thereby estimating the relative proportions of cell types. To investigate potential age-related differences in cellular composition, samples were stratified into young, middle, and elderly groups as defined above. We used the Wilcoxon rank-sum test to determine whether cell type proportions differed across age groups within each tissue.

**Inference of transcription factor activity**

We aimed to evaluate age-dependent changes in transcription factor (TF) activity and to visualize the distribution of cell type-specific TF activity. To this end, we independently inferred TF activity from the GTEx dataset (bulk RNA-seq data) and the Tabula Sapiens dataset (single-cell RNA-seq data). For both datasets, we employed the decoupleR framework (Badia-I-Mompel *et al.*, 2022) in combination with the Collection of Transcriptional Regulatory Interactions (CollecTRI) database, which provides a knowledge-based network of TF’s target gene interactions.

For the bulk RNA-seq data, we applied ordinal logistic regression to evaluate the association between gene expression levels and age groups. Specifically, the expression level of each gene was treated as the explanatory variable, and age group was modeled as the dependent variable, yielding a regression coefficient. This coefficient represents a statistical measure of age-dependent gene expression changes and was used as input for the decoupleR framework. We then inferred TF activity scores by integrating these statistics with the knowledge-based TF-target gene relationships provided by the CollecTRI database. A positive score indicates age-dependent TF activation, whereas a negative score implies TF repression.

For the single-cell RNA-seq data, to examine the cell type-specific distribution of TF activity, we also applied the decoupleR method to the Tabula Sapiens dataset. In this single-cell workflow, we used the gene expression profile of each cell as direct input to calculate the TF activity score for that cell. We performed uniform manifold approximation and projection (UMAP)-based dimensionality reduction on the single-cell TF activity scores to investigate their distribution across different cell types. This visualization enabled us to determine whether TFs identified as age-dependently activated (or repressed) in the bulk analysis were predominantly active in particular cell types, providing insights into transcriptional regulatory mechanisms associated with ageing.

To statistically assess differences in TF activity among cell types, we aggregated the TF activity scores and associated p-values for each cell type. We then performed Fisher’s multiple comparison test using the RVAideMemoire package in R (https://github.com/cran/RVAideMemoire). The following analyses were conducted for each cell type. First, TFs were grouped by regulation direction (up- or down-regulated). For each cell type, we counted TFs that exceeded a threshold (e.g., scaledscore > 0) with a p-value below a set cutoff, and organized these counts into a contingency table. Second, we applied Fisher’s exact test across cell types, correcting for multiple testing with the Benjamini-Hochberg method. For each cell type, we combined its pairwise p-values using the sumlog function in the metap package (https://github.com/cran/metap) and converted the result to a –log₁₀ scale. Finally, cell types were ranked by their –log₁₀ combined p-values to highlight the most enriched populations.

**Inference of cell-cell interaction**

We comprehensively evaluated ageing-associated changes in ligand-receptor (L-R) activity. First, we used BulkSignalR package (https://github.com/jcolinge/BulkSignalR) to analyse GTEx bulk RNA-seq data and identified L-R pairs displaying age-dependent expression patterns. Specifically, for each age group in the GTEx dataset, we estimated L-R pairs whose expression levels were correlated significantly with age. Next, we provided these age-specific L-R pairs, along with single-cell RNA-seq data from the Tabula Sapiens dataset, as input to CellChat package (https://github.com/jinworks/CellChat), which infers cell-cell communication networks. CellChat quantifies interactions between two cell populations mediated by a given ligand and its cognate receptor, assigns a probability value to each interaction, and performs permutation tests to determine biologically relevant cell-cell communication. We then calculated the overall strength of intercellular interactions by aggregating communication probabilities for each interaction and visualized these values using heatmaps. Additionally, the communication probabilities of individual L-R interactions were plotted as line graphs to illustrate their contribution to age-dependent cell-cell communication.

**References**

Badia-I-Mompel,P. *et al.* (2022) decoupleR: ensemble of computational methods to infer biological activities from omics data. *Bioinform Adv*, **2**, vbac016.

Guzz,P.H. *et al.* (2023) GTExVisualizer: a web platform for supporting ageing studies. *Bioinformatics*, **39**, btad303.

Jew,B. *et al.* (2020) Accurate estimation of cell composition in bulk expression through robust integration of single-cell information. *Nat. Commun.*, **11**, 1971.

Kryuchkova-Mostacci,N. and Robinson-Rechavi,M. (2017) A benchmark of gene expression tissue-specificity metrics. *Brief. Bioinform.*, **18**, 205–214.

Schneider,A.L. *et al.* (2024) voyAGEr, a free web interface for the analysis of age-related gene expression alterations in human tissues. *Elife*, **12**.

Yanai,I. *et al.* (2004) Genome-wide midrange transcription profiles reveal expression level relationships in human tissue specification. *Bioinformatics*, **21**, 650–659.
